# WACS: improving ChIP-seq peak calling by optimally weighting controls

**DOI:** 10.1186/s12859-020-03927-2

**Published:** 2021-02-15

**Authors:** Aseel Awdeh, Marcel Turcotte, Theodore J. Perkins

**Affiliations:** 1 School of Electrical Engineering and Computer Science, University of Ottawa, Ottawa, K1N6N5 Canada; 2 Regenerative Medicine Program, Ottawa Hospital Research Institute, Ottawa, K1H8L6 Canada; 3 Department of Biochemistry, Microbiology and Immunology, University of Ottawa, Ottawa, K1H8M5 Canada

**Keywords:** ChIP-seq, Controls, Bias

## Abstract

**Background:**

Chromatin immunoprecipitation followed by high throughput sequencing (ChIP-seq), initially introduced more than a decade ago, is widely used by the scientific community to detect protein/DNA binding and histone modifications across the genome. Every experiment is prone to noise and bias, and ChIP-seq experiments are no exception. To alleviate bias, the incorporation of control datasets in ChIP-seq analysis is an essential step. The controls are used to account for the background signal, while the remainder of the ChIP-seq signal captures true binding or histone modification. However, a recurrent issue is different types of bias in different ChIP-seq experiments. Depending on which controls are used, different aspects of ChIP-seq bias are better or worse accounted for, and peak calling can produce different results for the same ChIP-seq experiment. Consequently, generating “smart” controls, which model the non-signal effect for a specific ChIP-seq experiment, could enhance contrast and increase the reliability and reproducibility of the results.

**Result:**

We propose a peak calling algorithm, Weighted Analysis of ChIP-seq (WACS), which is an extension of the well-known peak caller MACS2. There are two main steps in WACS: First, weights are estimated for each control using non-negative least squares regression. The goal is to customize controls to model the noise distribution for each ChIP-seq experiment. This is then followed by peak calling. We demonstrate that WACS significantly outperforms MACS2 and AIControl, another recent algorithm for generating smart controls, in the detection of enriched regions along the genome, in terms of motif enrichment and reproducibility analyses.

**Conclusions:**

This ultimately improves our understanding of ChIP-seq controls and their biases, and shows that WACS results in a better approximation of the noise distribution in controls.

## Background

High throughput sequencing technologies help in uncovering the mechanisms of gene regulation and cell adaptation to external and internal environments [1, 2]. One widely used technology is chromatin immunoprecipitation followed by next generation sequencing (ChIP-seq). It allows the genome-wide investigation of the structural and functional elements encoded in a genomic sequence, such as transcriptional regulatory elements. The main goal of a ChIP-seq experiment is the detection of protein-DNA binding sites and histone modifications genome-wide in various cell lines and tissues. Many peak calling methods have been proposed for the identification of regions of enrichment (putative binding sites) in ChIP-seq data [3–7].

Every experiment is prone to noise and bias, and ChIP-seq experiments are no exception. While some read pileups correspond to regions of true enrichment, others may be a result of the distortion of the ChIP-seq signal. Biased or noisy datasets (with a high number of false negative or false positive peaks) negatively impact downstream biological and computational analyses [8]. Thus, accounting for both noise and bias is important. Existing peak callers generally account for noise by assessing statistical significance under some statistical model. Bias is a more complicated subject and is usually addressed explicitly only via some control data to which the ChIP-seq is compared. We return to the issue of controls shortly.

There are many sources of bias in a ChIP-seq experiment. In the experimental design, for example, the quality of the experiment is predetermined by antibody and immunoprecipitation specificity. Low sensitivity, resulting from poor affinity to the target protein of interest, or low specificity, from cross reactivity with other unrelated proteins, degrades the quality of a ChIP-seq experiment [9]. The fragmentation step may also introduce bias [10]. Prior to immunoprecipitation, the DNA-protein complexes undergo fragmentation. However, due to the non-uniform nature of the chromatin structure (DNA), some regions are more densely packed (heterochromatin) than others and are thus more resistant to fragmentation. Less densely packed regions (euchromatin) will undergo more fragmentation. Another source of bias is mappability, which is the extent to which reads are uniquely mapped to regions along the genome [10, 11]. In an ideal situation, long enough reads are used such that there is higher coverage and uniformity in coverage. However, in practice, read length is short and there are “ambiguous” reads that map to multiple regions. Such multiple mapped reads can either be retained (creating ambiguous ChIP-seq signal) or discarded (creating empty, unmappable regions), with either choice creating a different sort of bias. GC content bias [12, 13], introduced by PCR amplification or sequencing, also results in imbalanced coverage of reads along the genome. For example, in PCR amplification, both GC rich and GC poor fragments are underrepresented in sequencing data [12]. These variations in coverage can have a significant impact on the results obtained.

Systematic and experimental biases hinder the full potential of ChIP-seq analysis. Thus, the quality of the input samples is important, especially in large scale analysis where low quality datasets have greater effects [8, 14]. Consequently, more than a decade after ChIP-seq was introduced, the ENCODE and modENCODE consortia developed a set of ChIP-seq quality control metrics and guidelines to produce high quality reproducible data [9]. The protocols address all the stages of a ChIP-seq experiment, as bias and noise may be introduced at various stages, such as experimental design, execution, evaluation and storage methods [10].

One essential step for the alleviation of bias is the incorporation of control datasets in ChIP-seq analysis. It assists in the selection of true enrichment binding sites from false positives. Controls, such as input DNA and IgG, attempt to minimize the effects of immunoprecipitation, antibody imprecision, PCR-amplification, mappability bias, etc., and thereby increase the reliability of the results. In the input DNA, using the same conditions as the original ChIP-seq experiment, the DNA undergoes cross linkage and fragmentation. However, no antibody nor immunoprecipitation is used [9]. For the IgG control, sometimes referred to as a “mock” ChIP-seq experiment, all the same steps and conditions as the original ChIP-seq experiment are applied. However, a control antibody (not specific to the protein of interest) is adopted to interact with non-relevant genomic positions [9]. DNase-seq and ATAC-seq are used to tackle open chromatin regions. According to ENCODE [9], the input DNA and IgG controls should have a sequencing depth greater than or equal to the original ChIP-seq experiment. Higher sequencing depth is recommended since input DNA signals represent broader genomic chromatin regions than ChIP-seq [9, 10]. Other crucial factors addressed by the protocols include, but are not limited to, biological/technical replicates and library complexity.

Many existing peak calling algorithms allow testing enrichment compared to a control [7, 15–20]. Whether biases in controls and ChIP-seq data are the same is not known, however. None of these methods selects a control or estimates background signals. Depending on which controls are selected and their nature, peak callers can produce different results (i.e., binding site positions) for the same ChIP-seq experiment. The BIDCHIPS [21], CloudControl [22] and AIControl [23] studies have shown that different ChIP-seq datasets can be biased in different ways. They address different biases in different ChIP-seq datasets via the integration of multiple control datasets through regression to improve enrichment analysis. There are some limitations to these studies, however.

For example, BIDCHIPS [21] has the ability to re-prioritize peaks already identified by another peak calling method. However, only five notions of control are accounted for and there are no mechanisms for de novo peak calling based on the combined control [21]. The Hiranuma et al. [22, 23] studies prove the advantage of using more controls to model the background signal. In CloudControl [22], the controls are subsampled in their regression fit proportional to their weights. This then allows the single customized control to be used as input to any peak calling method. However, the downsampling of the combined controls may introduce noise into the control signal.

AIControl [23], a peak calling framework, is an extension of CloudControl [22]. It integrates a group of publicly available control datasets and uses ridge regression to model the background signal. This eliminates the need for the user to input controls. However, some users may want to provide their own controls, and this is not accommodated. Additionally, the number of datasets in ENCODE increases with time, so allowing controls as input in a weighted peak caller is important to represent the newly available datasets and newly explored cell lines.

In this work, we introduce a peak calling algorithm, Weighted Analysis of ChIP-Seq (WACS), which utilizes “smart” controls to model the non-signal effect for a specific ChIP-seq experiment. WACS first estimates the weights for each input control, without requiring the fine-tuning of any parameters. Using the weighted controls, WACS then proceeds to detect regions of enrichment along the genome. WACS is an extension of MACS2.1.1 (Model-based Analysis for ChIP-Seq) [18], the most highly cited open source peak caller. Our development of WACS based on MACS2 allows researchers to use the weighted approach within a peak calling method with which they are familiar, and which has many refined features. Fragment length estimation/detection, read shifting, candidate peak identification, and peak assessment remain the same, while the construction of the control via the weighted combination of datasets is different. To allow for potentially large numbers of controls, we restructure the code invisibly for better memory footprint. We also correct a hashing bug in the pileup-computing code of MACS2, which becomes especially important when we have high read depth and/or many controls. (This bug has subsequently been corrected in the main MACS2 distribution as well.)

We evaluate WACS on a large collection of 90 ChIP-seq datasets and 147 control datasets from the K562 cell line in the ENCODE database [24]. To establish generalizability and study performance in a less expansive setting, we also investigate WACS on 20 ChIP-seq datasets for each of the A549, GM12878 and HepG2 cell lines. (The terms ChIP-seq and treatment are used interchangeably throughout the paper.) We compare WACS to MACS2, as WACS is based on MACS2. We also compare WACS to AIControl, as it is the only other weighted peak caller which intellectually selects its controls. The results demonstrate the importance of smart bias removal methods and the use of customized control datasets for each ChIP-seq experiment, as the amount of bias varies across different ChIP-seq experiments. In the investigation of downstream genomic analysis, such as motif enrichment and reproducibility, the use of weighted controls in WACS shows a significant improvement in peak detection in comparison with the pooled unweighted controls in MACS2 and weighted controls in AIControl.

## Results

### WACS: a new algorithm for ChIP-seq peak calling with a weighted combination of controls

**Fig. 1:**
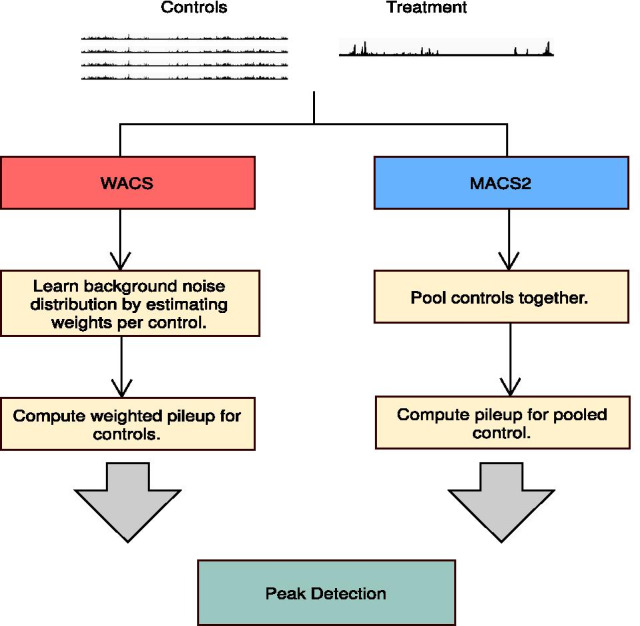
Flowcharts for WACS and MACS2. Both methods take controls and a treatment as input


Our approach, WACS, estimates a background distribution by weighting controls, and ultimately identifies regions of enrichment along the genome (Fig. 1 and Additional file 1: Figure S1). Below we describe the five major steps of the WACS algorithm. To implement WACS, we modified a well-known open source algorithm, MACS2. Because there is limited written description of how MACS2 works, we describe some parts of MACS2 to fully describe WACS. The WACS algorithm is summarized into two parts: Derive Weights (Algorithm 1) and Peak Detection (Algorithm 2).

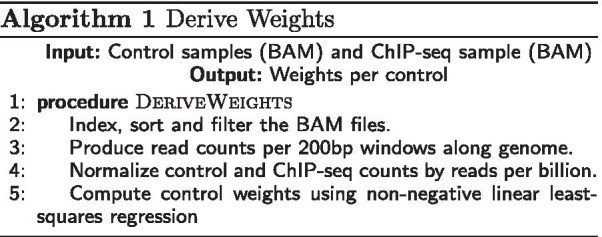

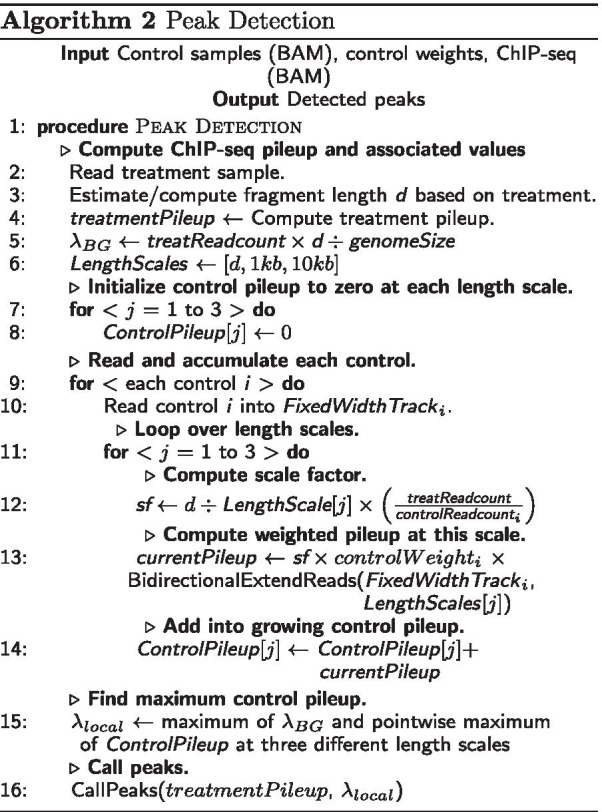


*Algorithm 1: Derive Weights.* The control and treatment samples (in BAM format) are first preprocessed, as seen in Algorithm 1. Using SAMtools [25], we index, sort and optionally filter (remove duplicates from) the BAM files (line 2 in Algorithm 1). We then use BEDtools [26] to convert the BAM files of mapped reads into read counts per 200 base pair (bp) windows along the genome with 50 bp increments (line 3 in Algorithm 1).

Next, WACS normalizes the mapped reads per window for the preprocessed control and treatment samples. This ensures that the control and treatment samples are on the same scale. WACS applies reads per billion normalization to both the control and ChIP-seq samples (line 4 in Algorithm 1). For each sample *m* and window *i*:

$$\begin{aligned} n_{mi} \leftarrow r_{mi}\ \times \ 10^9\ \div \ TotalReadCount_m \end{aligned}$$

where $r_{mi}$ is the read count in the window, $n_{mi}$ is the normalized read count, and $TotalReadCount_m$ is the total number of reads in sample *m*. This effectively reproduces the normalization in MACS2, which linearly scales the control sample to the ChIP-seq sample. In what follows, we assume *k* total controls comprise samples 1 to *k*, and sample *k* + 1 is the ChIP-seq data.

WACS then calculates the weights per input control (line 5 in Algorithm 1). WACS performs non-negative least squares (NNLS) to model the treatment dataset as a function of the controls. The overall objective of the regression is to find the values of the parameters (weights), that minimize the sum of squared differences between predictions and target values, with an additional constraint that allows only positive weights. Given *n* instances (windows), $y_i=n_{k+1,i}$ target values (one per window), $x_i=(n_{1i},\ldots ,n_{ki})$ feature vectors (one vector per window), a vector $\Theta$ of coefficient weights and a constant offset $\Theta _0$, NNLS’s objective function is:

$$\begin{aligned} \begin{aligned} \min _{\Theta ,\Theta _0} \frac{1}{2n} \sum _{i=1}^{n} (y_i - \Theta \cdot x_i - \Theta _0)^2 \\ \text {subject to } \Theta \ge 0 \\ \text {and } \Theta _0 \ge 0 \\ \end{aligned} \end{aligned}$$

To solve the NNLS regression we rely on the nnls module from scipy.optimize, part of the scipy [27] package in Python. This produces a weighted control model for the treatment, with weights that indicate the relative importance of each control in modelling the treatment background signal. Zero weights are given to controls not required for modelling the treatment experiment. If there is one control, WACS and MACS2 produce the same output, as by default, the control in WACS gets a weight of exactly 1. The controls can also be weighted by the user, instead of using NNLS to compute the weights of the controls.

*Algorithm 2: Peak Detection.* WACS is identical to MACS2 in its initial processing of the treatment sample, including: loading the mapped reads (line 2); estimation/calculation of fragment length *d*, which differs depending on whether the ChIP-seq reads are sequenced single-end or paired-end (line 3); and construction of the treatment pileup, which also differs for single-end or paired-end reads (line 4). Because these details have been described elsewhere, we do not repeat them here [18, 28, 29].

Where WACS differs substantially from MACS2 is how it reads in, processes, and combines the control samples. WACS reads the controls into memory one at a time, accumulating them into overall (weighted) control pileups at three different length scales: *d*, 1 kb and 10 kb. The length scale is essentially the diameter of a Parzen-windows density estimator used to smooth the control reads. As each control is read in, it is smoothed, scaled so that its total reads are commensurate with the treatment, and further scaled by the control weight computed in Algorithm 1 (unless the user opts for unweighted controls). The function BidirectionExtendReads performs the actual smoothing, extending the read starts into intervals with diameter equal to the length scale. The smoothed and scaled control is added to the growing overall control at that length scale. In contrast, MACS2 reads all the control data in before beginning smoothing, which can create an unmanageable memory footprint when very many controls are being combined. Finally, WACS (as does MACS) creates an overall control pileup by taking the pointwise maximum of the “background” read density $\lambda _{BG}$ and the control pileups computed at each length scale.

Finally, WACS calls peaks using the same mechanism as MACS2, which involves identifying candidate peaks and comparing the pileup heights at their summits with the control track. In the case of unweighted controls, WACS produces an identical control track to MACS2 and identical peak calls. However, when control samples are weighted differently, a different control track is produced and different peaks may be called. Each peak is associated with a p-value and a q-value, the latter accounting for multiple comparisons across the entire genome.

*Duplicate removal.* Duplicate reads—multiple reads mapped to the same position on the genome—are often due to the overamplification of DNA fragments by PCR, which leads to the repeated sequencing of a DNA fragment. For WACS and MACS2, duplicate removal is optional. To produce more reliable peak calls, MACS2/WACS remove redundant reads at each genomic locus for both the treatment and control datasets [18]. The default number per genomic locus is determined by the sequencing depth. However, when dealing with multiple controls, MACS2 performs duplicate removal after pooling reads. WACS does the same thing when used in unweighted mode, for the sake of consistency with MACS2. In this case, apparent “duplicates” arising from different sequencing runs may be removed incorrectly, artificially flattening the control read distribution in high density areas. This phenomenon can be particularly prominent when hundreds of controls are being pooled. Thus, we recommend that users who want to perform de-duplication do so prior to feeding the mapped read files to MACS2 or WACS.

### Average number of peaks per algorithm and average percentage overlap between algorithms

To evaluate the performance of WACS with other methods, we downloaded ChIP-seq and control data for four cell lines: K562, A549, GM12878 and HepG2. For each ChIP-seq sample, we generated peaks under five conditions: (1) MACS2 with all the controls from the same cell line (All MACS2), (2) MACS2 with the matched ENCODE controls (Matched MACS2), (3) WACS with all the controls from the same cell line (WACS), (4) WACS with 10 randomly selected controls from the same cell line (WACS Random10) and (5) AIControl with its predefined controls (AIControl). We also used two methods to study the quality of peaks. “All Peaks” considers all the original peaks output by each method, whilst “Standardized” peaks normalizes the peaks output by each ChIP-seq sample by the number of peaks and peak width. (See Methods below.)

In this section, we examine some basic statistics regarding the peaks generated by each algorithm and their corresponding pairwise overlap with the other peak calling methods. We focus on the K562 results in this and the following several subsections; results for additional cell lines are reported further below. This will help us understand how different the peak callers are. In Table 1, we report the average number of peaks output by each algorithm across the different ChIP-seq datasets for all peaks and standardized peaks.

**Table 1:** Average number of peaks

Type	WACS	WACS Random10	Matched MACS2	All MACS2	AIControl
All Peaks	12,457	17,239	24,422	26,892	91,113
Standardized	12,016	12,016	12,016	12,016	12,016

We notice that AIControl outputs the largest number of peaks—over seven times as many peaks as WACS, and nearly 4 times as many as Matched MACS2. WACS outputs the smallest number of peaks on average. Matched MACS2 and All MACS2 output approximately the same number of peaks, and roughly twice as many as produced by WACS, while WACS Random10 generates a number of peaks intermediate between WACS and MACS2. For standardized peaks, however, all algorithms have the same number of peaks per dataset, which averages out to 12016.

In Tables 2 and 3, we report the average percentage peak overlap between each pair of algorithms across the ChIP-seq datasets, for all peaks and standardized peaks respectively. More specifically, for every algorithm X (rows) and every other algorithm Y (columns), we computed the percentage of X’s peaks overlapping any of Y’s peaks for each of the 90 ChIP-seq datasets, and then averaged the percentages across the 90 datasets. When considering all peaks, for example, 27.1% of the peaks generated by WACS overlap with All MACS2 peaks. Most notably, less than 7% of the peaks generated by AIControl overlap with peaks generated by the other algorithms. For the other pairwise combinations, most overlaps are in the 30–40% range. Conversely, in Table 3 for standardized peaks, we notice an almost symmetrical matrix with an increase in percentage overlap across all algorithms, in comparison to Table 2. This is especially noticeable for AIControl, where approximately 25% of the AIControl peaks now overlap with peaks generated by other algorithms. All overlaps are in the range of 23–43%.

**Table 2:** Average percentage of all peaks overlapping

	WACS	WACS Random10	Matched MACS2	All MACS2	AIControl
WACS	–	37.3	29.0	27.1	29.0
WACS Random10	38.6	–	32.7	34.9	32.7
Matched MACS2	31.9	35.0	–	37.9	31.0
All MACS2	30.2	37.3	37.8	–	33.6
AIControl	6.5	6.3	5.0	5.5	–

**Table 3:** Average percentage of standardized peaks overlapping

	WACS	WACS Random10	Matched MACS2	All MACS2	AIControl
WACS	–	42.0	37.7	39.6	25.1
WACS Random10	42.0	–	39.5	42.7	25.0
Matched MACS2	37.7	39.4	–	41.7	23.5
All MACS2	39.6	42.7	41.2	–	24.3
AIControl	25.1	25.0	23.5	24.3	–

The different number of peaks generated by each algorithm, and the resultant differences in percentage overlaps, highlight the importance of standardizing the peaks to remove the effect of the number of peaks in our analysis. Standardizing the peaks allows us to select the top quality peaks for comparison.

### Peaks identified by WACS are more enriched for known sequence motifs

The purpose of ChIP-seq analysis is the identification of regions of enrichment, such as transcription factor (TF) binding sites, along the genome. Thus, DNA binding motifs for a TF tend to be enriched in genuine binding sites. To evaluate the performance of our method in comparison to MACS2 and AIControl, we performed motif enrichment analysis on the peaks. Adopting a similar method as in [23], we first used JASPAR to obtain position weight matrices (PWMs) for each unique TF [30]. Motifs in JASPAR are derived from in vitro assays, such as SELEX, and in vivo high throughput sequencing experiments, such as ChIP-seq or ChIP-exo [30]. (See Additional file 1: Table S5 for the PWM IDs per TF.) Using PWMs as input, we then used FIMO (Find Individual Motif Occurrences) [31] in the MEME suite [32] to scan the entire human genome GRCh38 and identify motif hits genome wide with a cutoff of 1e-5 to define significant matches. In our analysis, peaks with a motif are considered as true positives, while those lacking a motif hit are considered false positives. We quantify motif enrichment for a particular set of peaks as the precision, or equivalently the fraction of true positive peaks over total peaks.

**Fig. 2:**
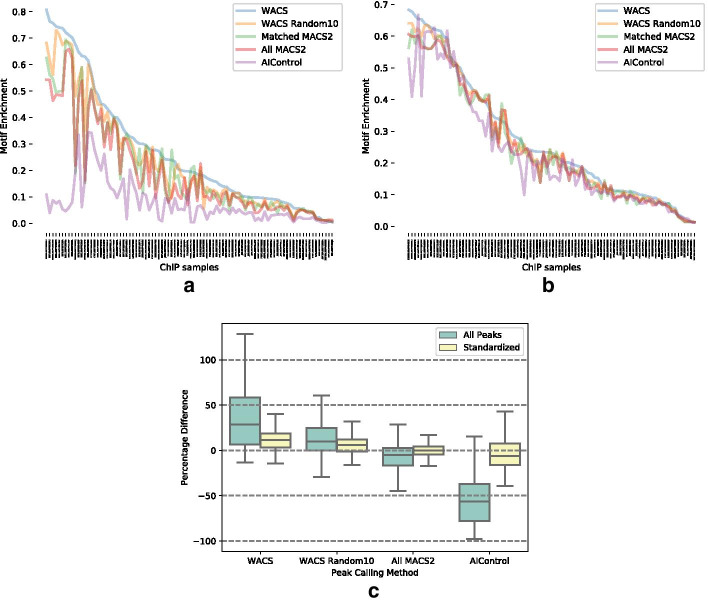
Motif enrichment of peaks found by five different peak calling approaches in 90 ChIP-seq samples. Motif enrichment is defined as the fraction of all peaks that contain at least one motif occurrence for the transcription factor in question. **a** Motif enrichment for all peaks. **b** Motif enrichment for the standardized peaks. **c** Distributions of percentages differences in motif enrichment relative to Matched MACS2. Box and whisker plots show the 0th, 25th, 50th, 75th and 100th percentiles

Figure 2a and b display the motif enrichment for each of the 90 ChIP-seq datasets for all peaks and standardized peaks respectively, when using WACS (blue line), WACS Random10 (yellow line), Matched MACS2 (green line), All MACS2 (red line) and AIControl (purple line). The ChIP-seq datasets have been sorted so that the WACS performance decreases from left to right. An immediate observation is that some ChIP-seq datasets result in much more motif-enriched peaks regardless of peak caller, while others have much less motif enrichment. This may have to do with factors such as specificity of the TF’s DNA binding, the accuracy of the JASPAR PWM used for motif search, or the quality of the ChIP-seq dataset itself.

When analyzing all the peaks (Fig. 2a), WACS is seen to outperform the other approaches the majority of the time—on 75 of 90 ChIP-seq samples in total. WACS Random10, All MACS2, and Matched MACS2 perform rather similarly, although we quantify this more carefully just below. AIControl performs the worst, with quite poor motif enrichment even in the datasets where all other algorithms perform very well. However, keeping in mind that AIControl tends to produce a large number of peaks, this could be a precision-recall sort of trade-off, in which the default behavior of AIControl is oriented towards the recall end of the spectrum. Indeed, when we examine the width- and number-standardized peaks (Fig. 2b), the performance of all algorithms is much more similar. We still see a strong effect that some ChIP-seq datasets have peaks with much better motif enrichment than others. We also still see that WACS still performs best, although by a smaller margin and less often—it is the top performer in 61 of 90 datasets. Table 4 reports the number of times out of 90 that each algorithm’s peaks show the best motif enrichment. By a proportion test, for either all or standardized peaks, WACS’s fraction of times as the top performer is statistically significantly greater than the expected fraction of 1/5 if all five algorithms performed equally well, with a p-value of less than $10^{-5}$. That WACS outperforms the other peak callers on the majority of the treatment samples even after standardization suggests that better motif enrichment is not a result of being more selective of the peaks, but that the peaks have inherently higher quality, at last as measured by motif enrichment.

**Table 4:** Number of datasets out of 90 where each algorithm’s peaks show the highest motif enrichment, compared to the other algorithms

Type	WACS	WACS Random10	Matched MACS2	All MACS2	AIControl
All peaks	75	5	7	3	0
Standardized	61	8	10	4	7

To evaluate further the quantitative differences in motif enrichment, we computed the percentage differences relative to Matched MACS2—which is the method used by ENCODE and something of a “gold standard”. Specifically, for each other algorithm and for each ChIP-seq dataset, we calculated the difference in motif enrichment, divided by the Matched MACS2 motif enrichment, and converted to a percentage. Figure 2c displays box plots of the percentages differences, for all peaks (green) and standardized peaks (yellow). For all the four methods, we observe that the standardized peaks in comparison to all peaks results in reduced dispersion and variability of the data. We will focus on standardized peaks in our discussion. For WACS, we notice a positive motif enrichment difference for most of the ChIP-seq datasets, with a mean improvement of 45% when all peaks are considered, or a more modest 14% when peaks are standardized. WACS Random10 also shows improvements over Matched MACS2 on average, although they are not as large as the WACS improvements. Nevertheless, all four cases (WACS and WACS Random10 with all or standardized peaks) are statistically significantly greater than zero by one-sample t-tests, with p-values of less than $10^{-5}$. All MACS2 performs similarly to Matched MACS2, as does AIControl when peaks are standardized, with none of the percent differences being statistically significantly different from zero. Without standardization, however, the full set of AIControl peaks is significantly worse on motif enrichment compared to Matched MACS2, with p-value less than $10^{-29}$. Overall, these results again confirm the improved performance of WACS compared to other approaches, although standardization reduces its advantage.

Another method for evaluating motif enrichment is the area under the precision-recall curve (AUPRC) [23]. The AURPC is designed to compare algorithms on the same set of instances. Each algorithm, however, generates a different set of peaks for a specific ChIP-seq dataset. Thus, we believe precision is a more appropriate evaluation metric than AUPRC for this comparison. Nevertheless, for the purpose of comparison with AIControl [23], which uses the AUPRC metric, we performed the AUPRC analysis as well. Additional file 1: Figure S2 shows an example precision-recall curve for the ChIP-seq dataset ENCFF109OWW with TF ZNF24, and Additional file 1: Figure S3 shows the the AUPRC for each of these ChIP-seq datasets when using standardized peaks. Using AUPRC, WACS outperforms WACS Random10, All MACS2, Matched MACS2 and AIControl on 73, 80, 78 and 81 of the 90 treatment samples respectively. These differences are statistically significant by a two-tailed sign test with p-value less than $10^{-5}$.

### Peaks identified by WACS are more reproducible

Ideally, a ChIP-seq peak calling algorithm is able to reproducibly identify true regions of enrichment along the genome with no false positives. Reproducibility is most commonly measured by computing the percentage overlap of peaks between replicates [4, 5]. As described above, the K562 experiments we chose included exactly two ChIP-seq biological replicate samples in 45 distinct experiments (see Additional file 1: Table S1). Using the five different peak calling approaches, we called peaks for every sample, and evaluated the overlap between replicate samples. Overlaps means we took one replicate and computed the fraction of peaks that overlaps with the other replicate.

**Fig. 3:**
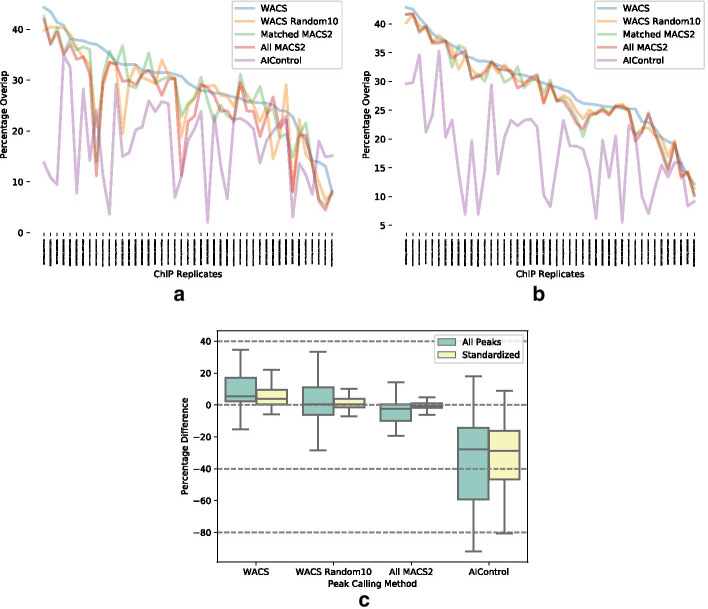
Reproducibility of peak calls between biological replicates. **a**, **b** Percentage overlap between replicates, for each of the five peak calling methods for 45 ChIP-seq experiments, when using **a** all peaks, or **b** standardized peaks. **c** Box plots of percentage difference in reproducibility relative to Matched MACS2

Figure 3a, b show the percentage overlap with all peaks and standardized peaks respectively for each of the ChIP-seq experiments when using WACS (blue line), WACS Random10 (yellow line), Matched MACS2 (green line), All MACS2 (red line) and AIControl (purple line). WACS has higher reproducibility than the other approaches on 26 of the 45 experiments when all peaks are considered, and on 28 of the 45 experiments with standardized peaks. These numbers are statistically significantly higher than expected under the null hypothesis that all algorithms perform equally, by a proportion test with p-value less than $10^{-4}$. AIControl has lowest reproducibility of the five approaches, regardless of whether all peaks or standardized peaks are considered. See Table 5 for details on all five algorithms.

**Table 5:** Number of experiments out of 45 for which each peak calling approach has the highest reproducibility between biological replicates

Type	WACS	WACS Random10	Matched MACS2	All MACS2	AIControl
All peaks	26	7	8	1	3
Standardized	28	7	5	4	1

To further investigate the quantitative differences in reproducibility, we computed the percentage differences in overlap relative to the overlap obtained by Matched MACS2. Figure 3c displays box plots of these percentage differences for all peaks (green) and standardized peaks (yellow). We notice a positive percentage difference in overlap for WACS, with 16% improved reproducibility for all peaks, or 5.6% for standardized peaks, on average. These differences are statistically significant by t-test with p-values of less than $10^{-3}$. However, WACS Random10’s performance is not statistically better than Matched MACS2, nor is All MACS2. AIControl has statistically significantly worse reproducibility both for all peaks ($p<0.05$) and standardized peaks ($p<10^{-12}$). The significance is borderline for the all peaks case, despite a large drop in mean, because of the high variability in its performance. Thus, in this section and the previous section, we see compelling evidence that WACS produces higher quality peaks than the other approaches, as measured by both motif enrichment and reproducibility between replicates.

### Controls used per treatment sample

Our results (and other results [21–23]) for motif enrichment and reproducibility analysis suggest that smart controls offer superior background subtraction and peak-calling for ChIP-seq data. However, the standard practice remains to generate controls alongside each ChIP-seq experiment, or to match them on the basis of experimental details, such as cell/tissue type, read length and sequencer. If smart controls are to be used, it is unclear how many controls should be considered, and how many will end up in the smart control. It is unclear whether ENCODE matched controls are, in fact, the best choices or even among the controls selected by a smart control procedure.

**Fig. 4:**
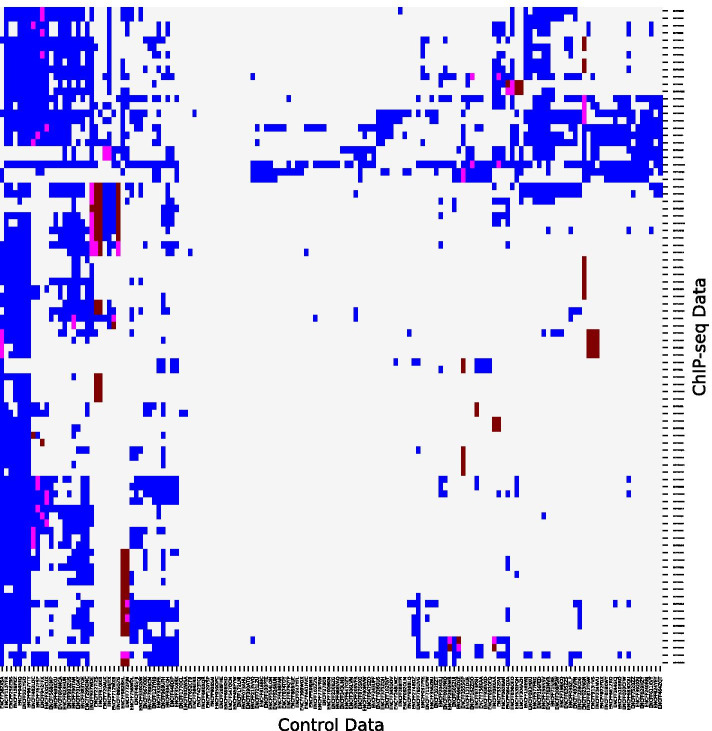
Comparison of controls used by WACS and ENCODE. The rows and columns correspond to the ChIP-seq and control experiments respectively. For each ChIP-seq dataset, the controls are given a *blue* color if they are used by WACS only, a *maroon* color if they are ENCODE matched controls only, and a *magenta* color if they are used by both ENCODE and WACS

Here, we aim to increase our understanding of the smart controls used to model the background signal. Figure 4 displays a matrix where the rows and columns represent the ChIP-seq and control datasets respectively. The blue color in the matrix represents the controls selected by WACS to fit each ChIP-seq dataset, the maroon color represents the ENCODE matched controls [24] and the magenta color represents the controls selected by both ENCODE and WACS.

Let us first consider the WACS selected controls per ChIP-seq dataset (blue) in Fig. 4. Different subsets of the 147 controls are required by WACS for each ChIP-seq dataset, but these form several coherent clusters, where groups of ChIP-seq datasets use relatively the same controls for modeling the background signal. For example, the 10 or so controls most towards the left of the diagram are used in modeling nearly all the ChIP-seq datasets’ backgrounds. The next 10 controls are widely used, though less so, and are distinct in be used for some of the ChIP-seqs towards the top. Conversely, there is a set of ChIP-seq datasets about near but not quite at the top of the matrix that rely on a large number of controls for modeling their background, whereas ChIP-seqs in the lower half rely almost solely on the leftmost controls.

Although each ChIP-seq’s background is modeled by a unique combination of controls, a clear trend is that many controls are combined—approximately 26 on average. Additional file 1: Figure S4 shows a histogram of the overall number of controls used by the ChIP-seq datasets using WACS.

For the ENCODE matched controls, we observe a range of 1 to 4 ENCODE matched controls per ChIP-seq dataset (maroon color in Fig. 4). For 40 of the 90 ChIP-seq datasets (44%), none of the matched ENCODE controls are used to model the background signal in comparison to those used by WACS (rows with no magenta color in Fig. 4). For example, 19 controls are used to model the background signal for the ChIP-seq dataset ENCFF651HPM in Fig. 4, none of which are the matched ENCODE controls. For the remaining 56% of the ChIP-seq datasets, some of the ENCODE matched controls are also those selected by WACS, as seen in Fig. 4 (magenta color), and there are 30 ChIP-seq datasets that use all their matched ENCODE controls (in addition to other controls samples). It is not clear from manual examination nor straightforward statistical analysis what features of a control, or jointly of a control and a ChIP-seq dataset, might cause the control to be desirable for inclusion. Determining the distinguishing characteristics of the best controls for a given ChIP-seq, beyond their utility in our regression formulation, is an important topic for future research.

Additionally, we further investigate which features resulted in the inclusion or exclusion of a control by WACS for a specific ChIP-seq dataset. An instance is defined as each control and ChIP-seq dataset combination, and the target value is a boolean which indicates whether that control was selected for that specific ChIP-seq dataset. For each instance, we consider boolean features representing the similarity or difference between the ChIP-seq and control datasets. These include lab name, experimental release year and mapped read length. A value of 1 indicates that the feature is equivalent for both the ChIP-seq and control datasets, and 0 otherwise. We conduct an exact Fisher’s test and found statistically significant results for each of these features with $p<0.005$. (See Additional file 1: Tables S7, S8 and S9). However, these predictions are far from perfect, and future work needs to be conducted to establish what a ‘good’ control is.

### Validation on additional cell lines

Here, we further evaluate WACS, MACS2 and AIControl on three other cell lines: A549, HepG2 and GM12878. We specifically explored 20 ChIP-seq and 18 control datasets for each cell line. (See Additional file 1: Tables S4, S5 and S6 for accession codes of the samples.) We evaluated MACS2 with the ENCODE matched controls (Matched MACS2), MACS2 with the cell line specific controls (All MACS2), WACS with the cell line specific controls (WACS), WACS with the all controls across the three different cell lines (WACS AllCtrls), and AIControl with its predefined set of controls on ChIP-seq datasets (AIControl).

**Fig. 5:**
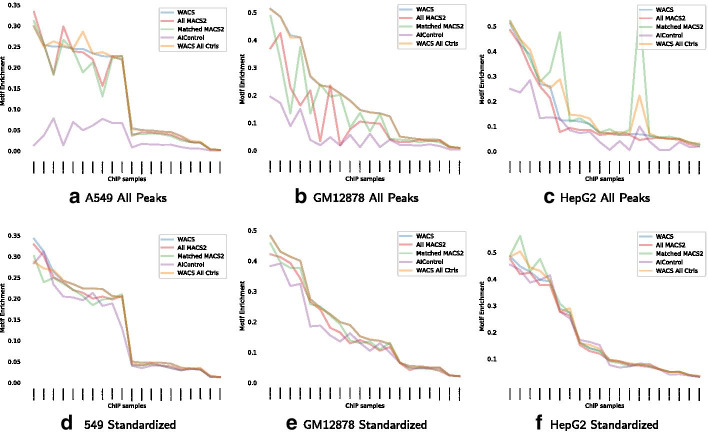
Motif enrichment of the peaks called by five methods for each of the three additional validation cell lines: A549 (**a**, **d**), GM12878 (**b**, **e**) and HepG2 (**c**, **f**)

To evaluate the quality of the peaks generated by each method for each cell line, we first investigate motif enrichment. Figure 5 displays the motif enrichment for all and standardized peaks for each of the ChIP-experiments corresponding to each cell line, when using WACS (blue line), WACS AllCtrls (yellow line), All MACS2 (red line), Matched MACS2 (green line) and AIControl (purple line). AIControl across all cell lines, for all and standardized peaks, has the lowest motif enrichment. For the cell line A549, as seen in Fig. 5a, d, WACS and WACS All Ctrls display the highest motif enrichment and have very similar performance. WACS and WACS All Ctrls outperform Matched MACS2, All MACS2 and AIControl on 14 treatment samples in total, as shown in Table 6. An equivalent trend is observed for the GM12878 cell line (Fig. 5b, e). However, when using all peaks, WACS has the highest motif enrichment; WACS outperforms WACS All Ctrls, Matched MACS2, All MACS2 and AIControl on 15 treatment samples in total, as shown in Table 6. Additionally, for standardized peaks, for cell lines A549 and GM12878, we notice almost equivalent motif enrichment when using All MACS2 and Matched MACS2. For HepG2 with all peaks (Fig. 5c), on the other hand, Matched MACS2 outperforms WACS, WACS All Ctrls, All MACS2 and AIControl on 11 treatment samples in total. For HepG2 with standardized peaks (Fig. 5f), all methods display similar performance.

**Table 6:** Numbers of datasets for which each algorithm produces peaks with the best motif enrichment

Cell Line	WACS	WACS AllCtrls	Matched MACS2	All MACS2	AIControl
*All peaks*					
A549	7	7	2	4	0
GM12878	15	2	2	1	0
HepG2	0	9	11	0	0
*Standardized*					
A549	10	8	1	1	0
GM12878	11	5	3	0	1
HepG2	3	6	5	0	6

**Fig. 6:**
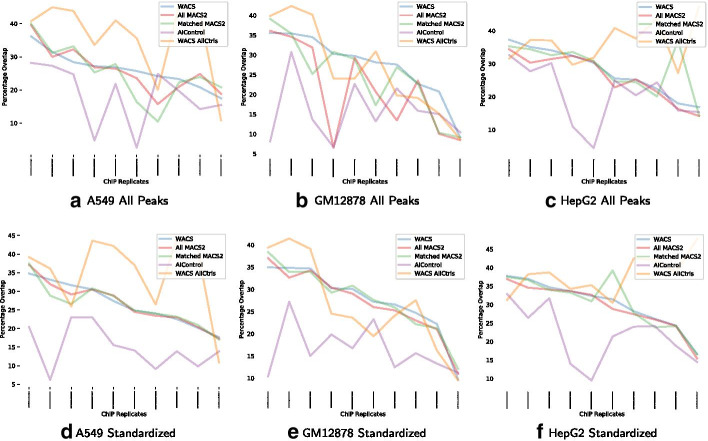
Percentage overlap in peaks between biological replicates, for each of the five peak calling methods for each of the three additional validation cell lines: A549 (**a**, **d**), GM12878 (**b**, **e**) and HepG2 (**c**, **f**)

Finally, we explore the reproducibility of peaks in ChIP-seq replicates for each cell line. There are a total of 10 ChIP-seq experiments for each cell line, each with two replicates. Figure 6 show the percentage overlap with all and standardized peaks for each of the ChIP-seq experiments, when using WACS (blue line), WACS All Ctrls (yellow line), All MACS2 (red line), Matched MACS2 (green line) and AIControl (purple line). WACS All Ctrls outperforms WACS, Matched MACS2, All MACS2 and AIControl on all of the ChIP-seq datasets for all the three cell lines, A549, GM12878 and HepG2 for all and standardized peaks, as show in Table 7. Again, AIControl displays the lowest percentage overlap for A549, GM12878 and HepG2 for all and standardized peaks.

**Table 7:** Number of datasets for which each algorithm produces peaks with the greatest overlap between biological replicates

Cell Line	WACS	WACS AllCtrls	Matched MACS2	All MACS2	AIControl
*All peaks*					
A549	0	8	1	0	1
GM12878	3	4	1	1	1
HepG2	1	7	2	0	0
*Standardized*					
A549	2	8	0	0	0
GM12878	2	4	3	1	0
HepG2	1	8	1	0	0

Moreover, we conduct a proportion test across all the three cell lines (A549, GM12878 and HepG2) for both motif enrichment and reproducibility. We notice that at times WACS outperforms the other peak calling methods, and other times WACS All Ctrls does. The high variability and small sample size leads to less significance when considering WACS or WACS All Ctrls separately. However, there is an overall positive favor in terms of WACS. For either all or standardized peaks, we observe that the fraction of datasets where both WACS and WACS All Ctrls have the highest motif enrichment and highest reproducibility is statistically significant with a p-value less than $10^{-5}.$

## Discussion

In this paper, we provide a method, WACS, for improved peak-calling and increase our understanding of ChIP-seq data, controls and their biases. WACS is built on the pre-existing, widely-used and precise peak calling method in MACS2, but has been recoded internally for better efficiency with many simultaneous datasets, and provides weights per control for a more accurate background model. We showed that this form of “intelligent” control construction is beneficial for peak calling. It appears to better estimate background signal in ChIP-seq datasets, as evidenced by better motif enrichment and better reproducibility in the called peaks. We showed that the controls selected by WACS are not necessarily the matched ENCODE controls. Additionally, for most of the ChIP-seq datasets, many more than two controls are selected to model the background signal. These findings run contrary to typical practice, where typically one or a small number of controls are chosen by the experimenter, sometimes based simply on controls having been done simultaneously with the ChIP-seq experiments, without any analysis of whether the control really models well the ChIP-seq background. As noted also by Hiranuma et al. [23], intelligent control selection or construction allows researchers to use other controls non-specific to their ChIP-seq experiment to model the noise distribution. This can decrease cost, time and resources required to perform the ChIP-seq experiments.

Moreover, WACS is a more selective peak caller in comparison to the other peak calling methods—as it outputs the least number of peaks on average. We evaluate WACS using all peaks and standardized peaks and observe that WACS outperforms the other peak calling methods in both cases. However, the difference in performance when using standardized peaks is less than when using all peaks. This shows that the performance of the other peak calling methods improves after standardization. Thus, this suggests that WACS pro-actively removes lower quality peaks.

Hiranuma et al. [23] claim that AIControl is better at removing background noise than MACS2. However, our results suggest the contrary. This may be due to a number of reasons. First, Hiranuma et al. [23] uses a different and nonstandard evaluation method for reproducibility analysis. Whereas we adopted the widely used approach of looking at peak overlaps between biological replicates [4, 5], Hiranuma showed that AIControl had higher irreproducibility than MACS when applied to unrelated datasets. Furthermore, Hiranuma et al. applied MACS2 using only one matched control, while for our analysis, we used either all the ENCODE matched controls for a treatment sample or simply all controls from the same K562 cell line. In either case, the provision of multiple controls may have improved MACS2’s performance.

In this manuscript, we described using NNLS to fit a model of ChIP-seq background to control densities, but other formulations are possible. For example, we experimented with an instance-weighted NNLS formulation, to account for differing variances on the regression targets $y_i$ (the ChIP-seq read counts per window). We did not find any improvement in performance. However, results may depend on how one estimates target variances. Relatedly, performing regression on log-transformed read counts may be worth exploring. RNA-seq analysis tools such as DESeq2 [33] use log linear models for read counts and comparisons between conditions. It would also make sense to explore L1-penalized regression formulations, to explore trade-offs between the number of controls used to model background and the accuracy of the background model.

Future work will deal with a more thorough analysis of the weighted controls approach on other high throughput sequencing data, such as RNA-seq, and other cell lines. The weighted approach will be used to study the biases in RNA-seq data across different platforms, labs, cell types, tissues, etc. For example, RNA-seq is used to measure the difference in gene expression between tissues, where a tissue consists of a mixture of cell types. To generate a realistic control tissue, the weighted approach can be used to weight the cell types in the tissue to model the background signal. Also, in this analysis, we focused on sharp peaks, which are more generally found at protein-DNA binding sites. Thus, an analysis of other broader peaks, for example, will be conducted. Ultimately, our overall aim is to increase the fidelity of conclusions drawn from high-throughput sequencing datasets, each of which may be biased in different ways, and to take fuller advantage of the masses of data already published as a “reference” for interpreting new data.

## Conclusion

We developed a peak calling method, WACS, which allows a mixture of weighted controls as input. The user inputs the controls. These controls can either be weighted by the user, or the weights can be computed by our regression approach. The latter systematically estimates the weights of the input controls to model the background signal for that ChIP-seq experiment. In the special case of equal weights which sum up to 1, the peaks output from WACS and MACS2 are identical. If different weights are allowed, the two algorithms have different outputs. WACS allows only positive weights for better interpretability of results. Negative weights are biologically difficult to interpret; as it does not add to the background signal. WACS proceeds to use this devised background signal to identify regions of enrichment along the genome. WACS is an extension of the most highly cited peak calling algorithm, MACS2 [18]. We conducted a comparison between WACS, MACS2 and AIControl to evaluate our method and the significance of the weighted controls. WACS significantly outperforms both MACS2 and AIControl in motif enrichment analysis and reproducibility analysis.

## Methods

We evaluated WACS, MACS2.1.1 (https://github.com/taoliu/MACS) and AIControl (https://github.com/hiranumn/AIControl.jl/) on data from the ENCODE consortium [34]. ENCODE ChIP-seq data is organized into “experiments”, which typically comprise two or more ChIP-seq samples generated at the same time and under the same conditions. Experiments also have controls matched to the ChIP-seq samples, and peaks called for each of the ChIP-seq samples. The K562 cell line has the most data available, so we focused our empirical evaluation on that data. We identified experiments with precisely two ChIP-seq samples. We included ChIP-seq BAM files mapped to the GRCh38 genome with filtered alignments. We further restricted attention to TFs with position-weight matrices in JASPAR. By these criteria, we identified 90 ChIP-seq samples (in 45 experiments) for analysis. We also collected all available controls for the K562 cell lines, resulting in 147 control samples for our analysis. Finally, to test the generality of our results in other cell lines, we selected 20 ChIP-seq and 18 control samples for each of A549, GM12878, and HepG2 cell lines. See Additional file 1: Tables S1, S2, S4, S5 and S6 for the accession codes of samples.

As seen in Fig. 1 (and Additional file 1: Figure S1), MACS2 pools the controls together for each ChIP-seq sample, whereas WACS estimates a weight for each control and computes a unique weighted control pileup for each ChIP-seq sample. AIControl uses a predefined set of publicly available controls [23]. We used two methods to evaluate the quality of the peaks generated by WACS, MACS2 and AIControl. One method considers all the original peaks output by each algorithm (called All Peaks). However, different peak callers can produce peaks in different locations based on the same data, and they can also produce different numbers of peaks. Thus, for additional comparison, we adopted the standardization procedure proposed by Hiranuma et al. [23], where the peak width and number of peaks are normalized for each treatment sample. First, the peak width is normalized by binning the peaks in 1000 base pair windows. For example, a peak at chromosome 1 from 14520 to 15420 is counted as two peaks covering bins 14000 to 15000 and 15000 to 16000. Next, the number of peaks for all five peak-calling conditions for the same dataset is normalized by retaining the top *n* most statistically significant peaks, where *n* is the smallest number of peaks in any of the five width-standardized peak sets.

## Supplementary Information

**Additional file 1: Table 1.** Table for the 45 ChIP-seq experiments and their corresponding ChIP-seq replicate samples and TFs for the K562 cell line from the ENCODE database used in our analysis. **Table 2**. Table for the 90 ChIP-seq samples and their corresponding control samples for the K562 cell line from the ENCODE database used in our analysis. **Table 3**. Table for the transcription factors (TFs) and their corresponding motif ID from JASPAR for 45 ChIP-seq experiments. **Table 4**. Table for the ChIP-seq experiments and their corresponding ChIP-seq replicate samples, TFs and controls for the A549 cell line from the ENCODE database used in our analysis. **Table 5**. Table for the ChIP-seq experiments and their corresponding ChIP-seq replicate samples, TFs and controls for the GM12878 cell line from the ENCODE database used in our analysis. **Table 6**. Table for the ChIP-seq experiments and their corresponding ChIP-seq replicate samples, TFs and controls for the HepG2 cell line from the ENCODE database used in our analysis. **Table 7**. Lab. **Table 8**. Year. **Table 9**. Mapped Read Length. **Figure 1**. Flowchart for the estimation of weights per control. **Figure 2**. Example of precision recall curve for TF ZNF24 ChIP-seq dataset ENCFF109OWW. **Figure 3**. AUPRC for the treatment samples. **Figure 4**. Histogram of the overall number of controls used per ChIP-seq dataset using WACS for 90 ChIP-seqs.

## Data Availability

ChIP-seq data used to develop and evaluate this method can be found online on the ENCODE website https://www.encodeproject.org. The WACS software can be found on the following website: https://www.perkinslab.ca/software.

## Abbreviations

WACS: Weighted Analysis of ChIP-seq
TF: Transcription factor
ChIP-seq: Chromatin immunoprecipitation followed by high throughput sequencing
NNLS: Non-Negative least squares
PWM: Position weight matrix
FIMO: Find individual motif occurrences
AUPRC: Area under the precision-recall curve
IQR: Interquartile range
