# Supplementary material for: WACS: improving ChIP-seq peak calling by optimally weighting controls
Source: BMC Bioinformatics. 2021 Feb 15;22:69. doi: 10.1186/s12859-020-03927-2 (PMC7885521; doi:10.1186/s12859-020-03927-2)
Supplement: Supplementary file 1 — Additional file 1: Table 1. Table for the 45 ChIP-seq experiments and their corresponding ChIP-seq replicate samples and TFs for the K562 cell line from the ENCODE database used in our analysis. Table 2. Table for the 90 ChIP-seq samples and their corresponding control samples for the K562 cell line from the ENCODE database used in our analysis. Table 3. Table for the transcription factors (TFs) and their corresponding motif ID from JASPAR for 45 ChIP-seq experiments. Table 4. Table for the ChIP-seq experiments and their corresponding ChIP-seq replicate samples, TFs and controls for the A549 cell line from the ENCODE database used in our analysis. Table 5. Table for the ChIP-seq experiments and their corresponding ChIP-seq replicate samples, TFs and controls for the GM12878 cell line from the ENCODE database used in our analysis. Table 6. Table for the ChIP-seq experiments and their corresponding ChIP-seq replicate samples, TFs and controls for the HepG2 cell line from the ENCODE database used in our analysis. Table 7. Lab. Table 8. Year. Table 9. Mapped Read Length. Figure 1. Flowchart for the estimation of weights per control. Figure 2. Example of precision recall curve for TF ZNF24 ChIP-seq dataset ENCFF109OWW. Figure 3. AUPRC for the treatment samples. Figure 4. Histogram of the overall number of controls used per ChIP-seq dataset using WACS for 90 ChIP-seqs. [file 12859_2020_3927_MOESM1_ESM.pdf]

Supplementary tables for:

Weighted analysis of ChIP-seq

Aseel Awdeh, Marcel Turcotte and Theodore J. Perkins

Table S1: Table for the 45 ChIP-seq experiments and their corresponding ChIP-seq replicate samples and TFs for the K562 cell line from the ENCODE database used in our analysis.

Experiment	Replicates	TF
ENCSR000BKQ	ENCFF401KIO, ENCFF398SXO	ETS1
ENCSR000BKT	ENCFF794ABP, ENCFF587WWS	USF1
ENCSR000BKU	ENCFF044TAL, ENCFF651HPM	YY1
ENCSR000BLI	ENCFF823GCX, ENCFF827SLL	E2F6
ENCSR000BMD	ENCFF331TRC, ENCFF535PLW	ELF1
ENCSR000BME	ENCFF784SCN, ENCFF778PDI	ZBTB7A
ENCSR000BMV	ENCFF492NUF, ENCFF561ILM	FOSL1
ENCSR000BMW	ENCFF204TUQ, ENCFF546IMN	REST
ENCSR000BNK	ENCFF454MMY, ENCFF310MOR	CTCFL
ENCSR000BRQ	ENCFF154YTN, ENCFF314OQP	CEBPB
ENCSR000DWE	ENCFF081HVQ, ENCFF494VZW	CTCF
ENCSR000EFS	ENCFF924CYX, ENCFF014UUB	JUN
ENCSR000EFV	ENCFF635HIS, ENCFF360QBV	MAX
ENCSR000EGI	ENCFF589IXE, ENCFF097GYE	MAFF
ENCSR000EGJ	ENCFF641HZR, ENCFF006GQZ	MYC
ENCSR000EGK	ENCFF168KTM, ENCFF299HHL	IRF1
ENCSR000EGN	ENCFF400BSN, ENCFF321ZQU	JUND
ENCSR000EGS	ENCFF239WGU, ENCFF836IMK	MYC
ENCSR000EGT	ENCFF489YJG, ENCFF728WOA	IRF1
ENCSR000EGZ	ENCFF726UVN, ENCFF388QNA	MXI1
ENCSR000EHE	ENCFF953ZZL, ENCFF729WGC	CEBPB
ENCSR000EWH	ENCFF771NSF, ENCFF078BWN	NR2C2
ENCSR000EWJ	ENCFF201BQU, ENCFF011OSI	E2F6
ENCSR000EWL	ENCFF613CDR, ENCFF156NIH	E2F4
ENCSR000EWN	ENCFF891GFG, ENCFF094FZQ	ZNF263

ENCSR000EZT	ENCFF749RRI, ENCFF784MLU	JUN
ENCSR000EZU	ENCFF014DRH, ENCFF553WMU	MYC
ENCSR000EZV	ENCFF357MHM, ENCFF491EUN	MYC
ENCSR000EZW	ENCFF814CHV, ENCFF050LIC	JUN
ENCSR000FAU	ENCFF089XMI, ENCFF263PLG	STAT1
ENCSR000FAV	ENCFF463LMJ, ENCFF403WNR	STAT1
ENCSR000FAZ	ENCFF439NNR, ENCFF483RLD	MYC
ENCSR041AXL	ENCFF871KNP, ENCFF490ICL	RFX1
ENCSR099NCH	ENCFF205VDU, ENCFF109OWW	ZNF24
ENCSR486IFJ	ENCFF857YYV, ENCFF320WXN	ESRRA
ENCSR494TDU	ENCFF337GMY, ENCFF821HMU	NRF1
ENCSR508DQA	ENCFF563FZR, ENCFF373AFZ	FOXK2
ENCSR563LLO	ENCFF846CYU, ENCFF226KOW	E2F1
ENCSR588AKU	ENCFF011XRF, ENCFF812KIP	RUNX1
ENCSR795IYP	ENCFF512YJL, ENCFF090SFR	JUNB
ENCSR819LHG	ENCFF364SJK, ENCFF611PXX	FOXA1
ENCSR837EYC	ENCFF564KCD, ENCFF207NLX	NRF1
ENCSR876GXA	ENCFF632WSK, ENCFF226IAA	ZBTB33
ENCSR968GIB	ENCFF837ZOY, ENCFF183MSQ	RFX1
ENCSR998AJK	ENCFF722LJA,ENCFF564CXM	NRF1

Table S2: Table for the 90 ChIP-seq samples and their corresponding control samples for the K562 cell line from the ENCODE database used in our analysis.

ChIP-seq	Controls
ENCFF310MOR	ENCFF772PJM, ENCFF982BHL
ENCFF263PLG	ENCFF332CUX
ENCFF109OWW	ENCFF895QZG, ENCFF227IZS, ENCFF910IKB, ENCFF937WDE
ENCFF953ZZL	ENCFF023NGN
ENCFF439NNR	ENCFF767FSP
ENCFF589IXE	ENCFF023NGN
ENCFF183MSQ	ENCFF712WXB, ENCFF790TAN
ENCFF491EUN	ENCFF332CUX
ENCFF094FZQ	ENCFF355SGP
ENCFF827SLL	ENCFF812TGW, ENCFF234NVU, ENCFF913HVS, ENCFF382XSA
ENCFF078BWN	ENCFF355SGP
ENCFF611PXX	ENCFF796JTX, ENCFF720AUK
ENCFF641HZR	ENCFF023NGN
ENCFF226KOW	ENCFF895QZG, ENCFF227IZS, ENCFF910IKB, ENCFF937WDE
ENCFF097GYE	ENCFF023NGN
ENCFF239WGU	ENCFF942FFX
ENCFF492NUF	ENCFF772PJM, ENCFF982BHL
ENCFF564KCD	ENCFF709XAA, ENCFF332SVJ
ENCFF201BQU	ENCFF355SGP
ENCFF044TAL	ENCFF772PJM, ENCFF982BHL
ENCFF836IMK	ENCFF942FFX
ENCFF635HIS	ENCFF023NGN
ENCFF398SXO	ENCFF772PJM, ENCFF982BHL
ENCFF204TUQ	ENCFF772PJM, ENCFF982BHL
ENCFF561ILM	ENCFF772PJM, ENCFF982BHL
ENCFF651HPM	ENCFF772PJM, ENCFF982BHL
ENCFF331TRC	ENCFF812TGW, ENCFF234NVU, ENCFF913HVS, ENCFF382XSA
ENCFF050LIC	ENCFF482LDC
ENCFF360QBV	ENCFF023NGN
ENCFF546IMN	ENCFF772PJM, ENCFF982BHL
ENCFF553WMU	ENCFF482LDC
ENCFF821HMU	ENCFF227IZS, ENCFF910IKB
ENCFF400BSN	ENCFF023NGN

ENCFF403WNR	ENCFF767FSP
ENCFF512YJL	ENCFF162ZOO, ENCFF332SVJ
ENCFF337GMY	ENCFF227IZS, ENCFF910IKB
ENCFF778PDI	ENCFF772PJM, ENCFF982BHL
ENCFF784SCN	ENCFF772PJM, ENCFF982BHL
ENCFF299HHL	ENCFF942FFX
ENCFF729WGC	ENCFF023NGN
ENCFF564CXM	ENCFF227IZS, ENCFF910IKB
ENCFF784MLU	ENCFF942FFX
ENCFF846CYU	ENCFF895QZG, ENCFF227IZS, ENCFF910IKB, ENCFF937WDE
ENCFF156NIH	ENCFF355SGP
ENCFF494VZW	ENCFF873PSH
ENCFF205VDU	ENCFF895QZG, ENCFF227IZS, ENCFF910IKB, ENCFF937WDE
ENCFF871KNP	ENCFF227IZS, ENCFF910IKB
ENCFF321ZQU	ENCFF023NGN
ENCFF728WOA	ENCFF482LDC
ENCFF154YTN	ENCFF304AZH, ENCFF984QXA, ENCFF533FQH, ENCFF836OEO
ENCFF089XMI	ENCFF332CUX
ENCFF454MMY	ENCFF772PJM, ENCFF982BHL
ENCFF011OSI	ENCFF355SGP
ENCFF794ABP	ENCFF772PJM, ENCFF982BHL
ENCFF613CDR	ENCFF355SGP
ENCFF081HVQ	ENCFF873PSH
ENCFF168KTM	ENCFF942FFX
ENCFF837ZOY	ENCFF712WXB, ENCFF790TAN
ENCFF483RLD	ENCFF767FSP
ENCFF891GFG	ENCFF355SGP
ENCFF226IAA	ENCFF895QZG, ENCFF227IZS, ENCFF910IKB, ENCFF937WDE
ENCFF090SFR	ENCFF162ZOO, ENCFF332SVJ
ENCFF006GQZ	ENCFF023NGN
ENCFF563FZR	ENCFF895QZG, ENCFF227IZS, ENCFF910IKB, ENCFF937WDE
ENCFF857YYV	ENCFF895QZG, ENCFF227IZS, ENCFF910IKB, ENCFF937WDE
ENCFF722LJA	ENCFF227IZS, ENCFF910IKB
ENCFF924CYX	ENCFF023NGN
ENCFF823GCX	ENCFF812TGW, ENCFF234NVU, ENCFF913HVS, ENCFF382XSA
ENCFF490ICL	ENCFF227IZS, ENCFF910IKB
ENCFF011XRF	ENCFF285EWB, ENCFF696ZGZ, ENCFF709XAA

ENCFF314OQP	ENCFF304AZH, ENCFF984QXA, ENCFF533FQH, ENCFF836OEO
ENCFF489YJG	ENCFF482LDC
ENCFF726UVN	ENCFF023NGN
ENCFF771NSF	ENCFF355SGP
ENCFF535PLW	ENCFF812TGW, ENCFF234NVU, ENCFF913HVS, ENCFF382XSA
ENCFF207NLX	ENCFF709XAA, ENCFF332SVJ
ENCFF814CHV	ENCFF482LDC
ENCFF373AFZ	ENCFF895QZG, ENCFF227IZS, ENCFF910IKB, ENCFF937WDE
ENCFF632WSK	ENCFF895QZG, ENCFF227IZS, ENCFF910IKB, ENCFF937WDE
ENCFF587WWS	ENCFF772PJM, ENCFF982BHL
ENCFF388QNA	ENCFF023NGN
ENCFF357MHM	ENCFF332CUX
ENCFF401KIO	ENCFF772PJM, ENCFF982BHL
ENCFF014DRH	ENCFF482LDC
ENCFF364SJK	ENCFF796JTX, ENCFF720AUK
ENCFF320WXN	ENCFF895QZG, ENCFF227IZS, ENCFF910IKB, ENCFF937WDE
ENCFF749RRI	ENCFF942FFX
ENCFF014UUB	ENCFF023NGN
ENCFF463LMJ	ENCFF767FSP
ENCFF812KIP	ENCFF285EWB, ENCFF696ZGZ, ENCFF709XAA

Table S3: Table for the transcription factors (TFs) and their corresponding motif ID from JASPAR for 45 ChIP-seq experiments.

TF	ID
MXI1	MA1108.1
E2F4	MA0470.1
MAFF	MA0495.1
CEBPB	MA0466.1
JUNB	MA0490.1
CTCFL	MA1102.1
YY1	MA0095.2
USF1	MA0093.2
REST	MA0138.2
ESRRA	MA0592.1
ELF1	MA0473.1
STAT1	MA0137.2
ZNF24	MA1124.1
ZBTB7A	MA0750.2
IRF1	MA0050.2
JUN	MA0488.1
CTCF	MA0139.1
NR2C2	MA0504.1
NRF1	MA0506.1
FOXA1	MA0148.1
FOXK2	MA1103.1
JUND	MA0491.1
E2F1	MA0024.2
E2F6	MA0471.1
ZNF263	MA0528.1
ZBTB33	MA0527.1
RUNX1	MA0002.2
MAX	MA0058.2
FOSL1	MA0477.1

Table S4: Table for the ChIP-seq experiments and their corresponding ChIP-seq replicate samples, TFs and controls for the A549 cell line from the ENCODE database used in our analysis.

Experiment	Replicates	TF	Controls
ENCSR182OZC	ENCFF791DRP<br>ENCFF217RBI	CEBPB	ENCFF634ULC<br>ENCFF632UPH<br>ENCFF368OTV
ENCSR375BUB	ENCFF073MBT<br>ENCFF280ZFT	CEBPB	ENCFF214UMU<br>ENCFF773DUX<br>ENCFF408NFU
ENCSR606ZTC	ENCFF347MNU<br>ENCFF417GPF	CEBPB	ENCFF455UAB<br>ENCFF887YTT<br>ENCFF081TBO
ENCSR623KNM	ENCFF757GXN<br>ENCFF826GGN	ELK1	ENCFF949XNJ<br>ENCFF918AJW
ENCSR000BQO	ENCFF125MJO<br>ENCFF585INN	FOSL2	ENCFF656HEF
ENCSR419TWL	ENCFF504YVD<br>ENCFF595EIS	HES2	ENCFF634ULC<br>ENCFF632UPH<br>ENCFF368OTV
ENCSR991VVW	ENCFF476XBN<br>ENCFF761UEZ	JUN	ENCFF193ABY<br>ENCFF222ACA<br>ENCFF639UDD
ENCSR269RPR	ENCFF179XAQ<br>ENCFF330XFU	JUNB	ENCFF171YYX<br>ENCFF631DES<br>ENCFF298EPS
ENCSR431LRW	ENCFF599JTK<br>ENCFF389OFH	JUNB	ENCFF653HKQ<br>ENCFF097CSC<br>ENCFF987XCE
ENCSR892DRK	ENCFF364NWO<br>ENCFF808ADX	REST	ENCFF572IKT<br>ENCFF714AMB

Table S5: Table for the ChIP-seq experiments and their corresponding ChIP-seq replicate samples, TFs and controls for the GM12878 cell line from the ENCODE database used in our analysis.

Experiment	Replicates	TF	Controls
ENCSR841NDX	ENCFF028TNY<br>ENCFF444PPF	ELF1	ENCFF666ATR<br>ENCFF322NTO
ENCSR000BMB	ENCFF739SRY<br>ENCFF735DGJ	ELF1	ENCFF488YYE
ENCSR000DZB	ENCFF211VKF<br>ENCFF784XUE	ELK1	ENCFF477ZKJ<br>ENCFF450WED<br>ENCFF824NQO<br>ENCFF710SMS<br>ENCFF579QDW<br>ENCFF813LMQ
ENCSR000BGY	ENCFF240MQI<br>ENCFF888PAI	IRF4	ENCFF562HPN<br>ENCFF100EIH<br>ENCFF438FFV
ENCSR000BQL	ENCFF983YCI<br>ENCFF207QTV	NFATC1	ENCFF754WTG<br>ENCFF966AVZ<br>ENCFF537DAJ
ENCSR000BGR	ENCFF791EPM<br>ENCFF845MYC	PBX3	ENCFF562HPN<br>ENCFF100EIH<br>ENCFF438FFV
ENCSR000BQS	ENCFF894EID<br>ENCFF569QEN	REST	ENCFF430ZCF<br>ENCFF100EIH<br>ENCFF438FFV<br>ENCFF562HPN
ENCSR000BRI	ENCFF884LEJ<br>ENCFF579PRC	RUNX3	ENCFF754WTG<br>ENCFF966AVZ<br>ENCFF537DAJ
ENCSR000BGE	ENCFF263NOT<br>ENCFF731ZNW	SRF	ENCFF862QZT<br>ENCFF289ONG
ENCSR000BGI	ENCFF737VAT<br>ENCFF074OYP	USF	ENCFF862QZT<br>ENCFF289ONG

Table S6: Table for the ChIP-seq experiments and their corresponding ChIP-seq replicate samples, TFs and controls for the HepG2 cell line from the ENCODE database used in our analysis.

Experiment	Replicates	TF	Controls
ENCSR000BQI	ENCFF090KCF<br>ENCFF499BWX	CEBPB	ENCFF175NMQ<br>ENCFF285LVE
ENCSR000EEE	ENCFF677PSB<br>ENCFF514FUP	CEBPB	ENCFF165KZY
ENCSR000BIE	ENCFF435DKZ<br>ENCFF178SXE	CTCF	ENCFF175NMQ<br>ENCFF285LVE
ENCSR112ALD	ENCFF011HOS<br>ENCFF320SCI	CREB1	ENCFF950AXC<br>ENCFF190EPQ
ENCSR267DFA	ENCFF396NXZ<br>ENCFF988UCQ	FOXA1	ENCFF950AXC<br>ENCFF190EPQ
ENCSR000BMO	ENCFF332SRJ<br>ENCFF401YVR	FOXA1	ENCFF175NMQ<br>ENCFF193YIO<br>ENCFF285LVE<br>ENCFF943DZB
ENCSR000BHP	ENCFF953PCA<br>ENCFF185DVY	FOSL2	ENCFF943DZB<br>ENCFF193YIO
ENCSR000BMZ	ENCFF930EXY<br>ENCFF418EVV	ELF1	ENCFF175NMQ<br>ENCFF285LVE
ENCSR000BJL	ENCFF195HUS<br>ENCFF291BBG	REST	ENCFF249PQD<br>ENCFF741TQN
ENCSR000EEK	ENCFF074GYD<br>ENCFF122BQB	JUN	ENCFF165KZY

Table S7: Lab

Features	Controls Used	Controls Not Used	Row Total
Same Lab	376	2208	2584
Not Same Lab	1709	8397	10106
Column Total	2085	10605	12690

Table S8: Year

Features	Controls Used	Controls Not Used	Row Total
Same Year	617	1945	2562
Not Same Year	1468	8660	10128
Column Total	2085	10605	12690

Table S9: Mapped Read Length

Features	Controls Used	Controls Not Used	Row Total
Same Length	375	1541	1916
Not Same Length	1710	9064	10774
Column Total	2085	10605	12690

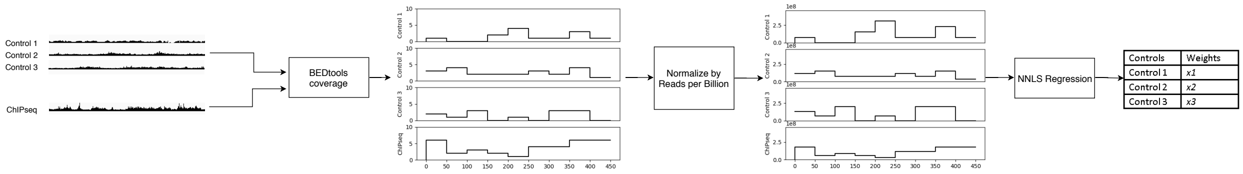

Figure S1: Flowchart for the estimation of weights per control.

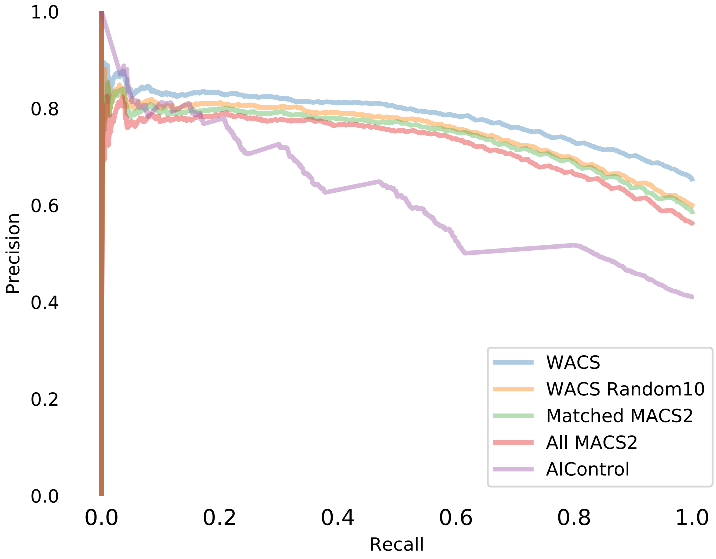

Figure S2: Example of precision recall curve for TF ZNF24 ChIP-seq dataset ENCFF109OWW.

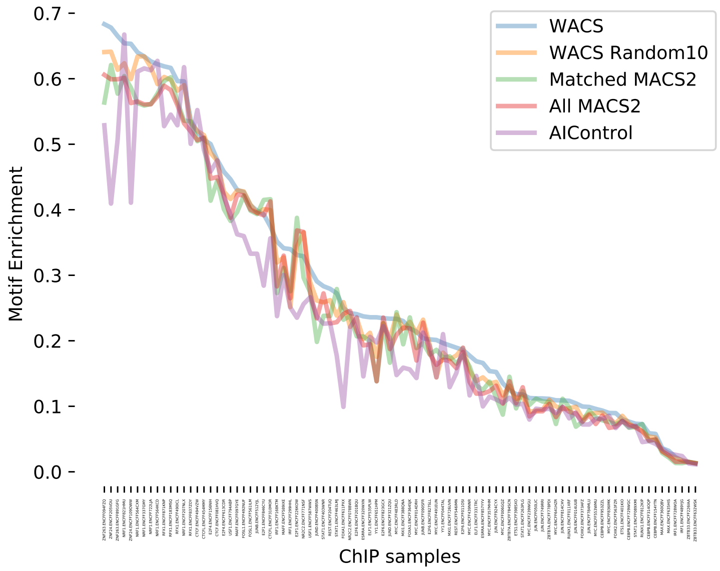

Figure S3: AUPRC for the treatment samples.

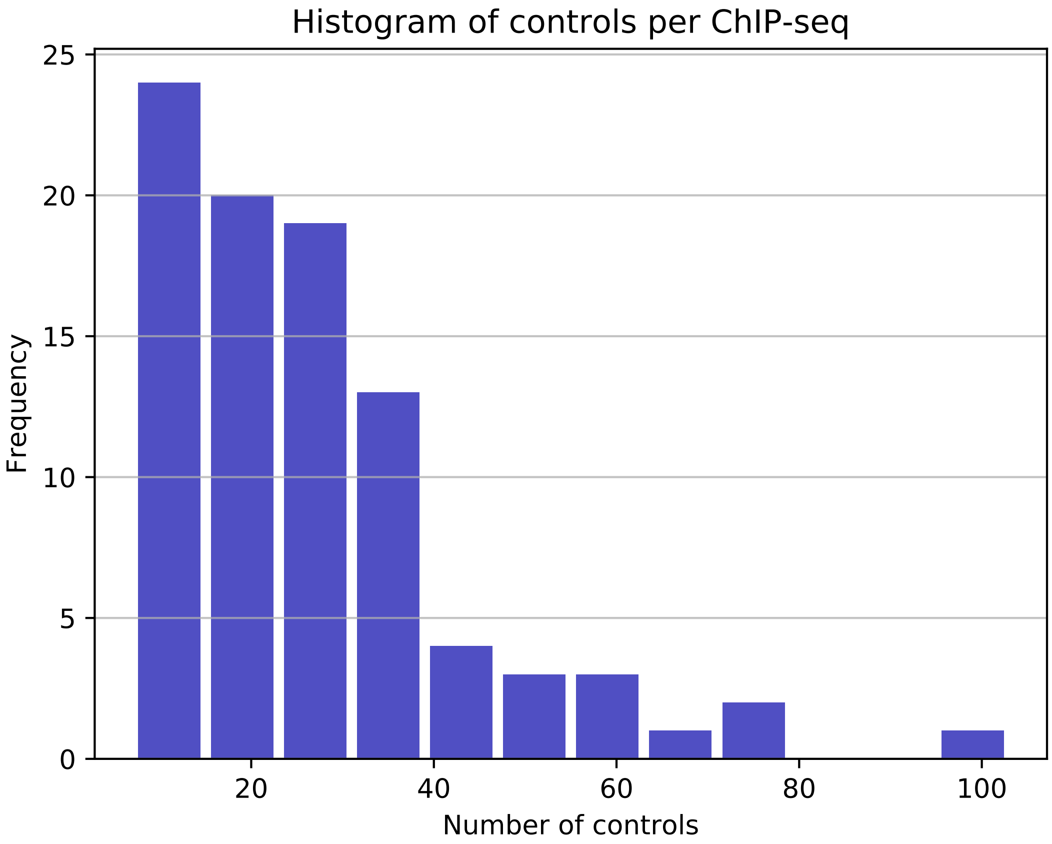

Figure S4: Histogram of the overall number of control used per ChIP-seq dataset using WACS for 90 ChIP-seqs.
